# Diversity of lactic acid bacteria in vegetable-based and meat-based fermented foods produced in the central region of Vietnam

**DOI:** 10.3934/microbiol.2017.1.61

**Published:** 2017-02-07

**Authors:** Yen Thi Ngoc Phan, Minh Thuy Tang, Tu Thi Minh Tran, Van Huu Nguyen, Trang Hien Nguyen, Takeshi Tsuruta, Naoki Nishino

**Affiliations:** 1 Graduate School of Life and Environmental Science, Okayama University, Okayama 700-8530, Japan; 2 Faculty of Animal Science, Hue University of Agriculture and Forestry, 102 Phung Hung, Hue, Vietnam; 3 Faculty of Engineering and Food Technology, Hue University of Agriculture and Forestry, 102 Phung Hung, Hue, Vietnam

**Keywords:** fermentation, lactic acid bacteria, meat, vegetable, Vietnam

## Abstract

The diversity of lactic acid bacteria (LAB) in naturally fermented foods produced in Hue, a city in the central region of Vietnam, was examined. From local markets, a total of 25 samples of three vegetable-based fermented products, specifically *dua gia* (bean sprouts), *dua cai* (cabbage), and *mang chua* (bamboo shoots), and two meat-based fermented products, specifically *nem chua* (uncooked pork) and *tre* (cooked pork) were obtained. The LAB diversity was assessed by quantitative real-time polymerase chain reaction (PCR) and qualitative denaturing gradient gel electrophoresis. Lactic and acetic acid contents were greater in meat-based products than in vegetable-based products. Major LAB species found in vegetable-based products (*Lactobacillus plantarum, Lactobacillus fermentum*, and *Lactobacillus helveticus*) were different from those identified in meat-based products (*Pediococcus pentosaceus*, *Weissella cibaria*, and *Lactococcus lactis*). The total bacterial population was approximately 10^9−10^ copies/g regardless of the food item, with the proportion of *Lactobacillus* spp. determined to be from 78% (*dua cai*) to 94% (*nem chua*).

## 1. Introduction

Most East Asian fermented foods are non-dairy products including various raw materials such as cereals, soybeans, fruits, and vegetables, as well as fish and other marine products. These foods are produced largely in households or on a small scale using local indigenous microbiota; this is different from western countries in which fermented foods are industrially produced on a large scale using selected starter cultures [1],[2].

Many vegetables such as cabbage, eggplant, beansprouts, carrots, bamboo shoots, scallions, and cauliflower can be used for fermentation. Common dishes from Vietnam include *dua gia*, *dua cai*, and *mang chua*, which are fermented beansprouts, cabbage, and bamboo shoots, respectively. Although the recipes can vary among households and commodities, the process consists of several basic steps. The roots of fresh plants are removed and the greens are washed and put in a basket to dry in the sun for three to four hours. The materials are then submerged in brine made from salt and sugars. Various other ingredients such as garlic, onion, chili, ginger, and young galangals can be added according to the recipe. The fermentation typically lasts for about two or three days at ambient temperature which can vary from 25 °C to 35 °C [3].

Fermented meat is also a standard dish and *nem chua* is consumed throughout Vietnam. Its main ingredients include finely ground pork lean (55–60%), boiled and sliced pork rind (30–35%), and other ingredients such as ground roasted rice, sugar, salt, and spices. The meat paste is shaped into small cubes or cylinders, covered with guava leaves or star gooseberry leaves, and then wrapped in banana leaves by folding the two ends of the rolls [4]. This careful wrapping is to prevent air penetration into the mixture, thus creating a relatively anaerobic environment that favours the growth of lactic acid bacteria (LAB) and inhibits the growth of potential pathogens. Fermentation takes place for two to four days at ambient temperature and the resulting product has a shelf-life of five days when preserved at room temperature [2],[3]. *Tre* is another type of fermented meat produced and consumed in the central region of Vietnam. In addition to pork lean and rind, the ear and nose of the pig are often included. Likewise, the meat mixture is subjected to heat treatment (grilling) before blending with rice and other vegetable ingredients.

Several attempts have been made to characterize the LAB microbiota in Vietnamese fermented foods. Nguyen et al described LAB microbiota associated with vegetable-based fermented foods produced in northern Vietnam [3]. Major LAB species isolated from *dua muoi* (mustard), *cu cai muoi* (beet), and *ca muoi* (eggplant) were *Lactobacillus plantarum, Lactobacillus fermentum*, and *Lactobacillus pentosus*. *L. fermentum* was prevalent in *dua muoi* (mustard) and *cu cai muoi* (beet) and *L. pentosus* was predominantly found in *ca muoi* (eggplant). Regarding meat-based fermented food, Tran et al. examined the LAB microbiota isolated from *nem chua* (uncooked pork) produced in northern, central, and southern Vietnam [4]. Although *L. plantarum* and *P. pentosaceus* were two major LAB species, independent of regional variations, *L. plantarum* was more often found in the northern and southern regions, whereas *P. pentosaceus* was predominant in the central region [4]. Nguyen et al. also reported the diversity of LAB isolates in *nem chua* (uncooked pork) produced in the northern region; *Lactobacillus farciminis* was detected more frequently than *L. plantarum*, whereas *Leuconostoc citreum*, *L. pentosus*, and *Lactobacillus brevis* were detected more frequently than *P. pentosaceus* [3].

Despite regional differences observed for LAB microbiota [4], most studies examined products in the north (Ha Noi) and south (Ho Chi Minh City) and few examined those in the central region of Vietnam. Because *nem chua* (uncooked pork) and *tre* (cooked pork) are different regarding the use of heat processing before fermentation, these two food items, consumed in the central region, are worth comparing. Likewise, most findings regarding LAB microbiota were derived from de Man, Rogosa, Sharpe (MRS) isolates; hence, more diversified microbiota could be observed if analysis was independent of plate-culture. In this study, three vegetable-based and two meat-based fermented foods were obtained from local markets and households in Hue, a city in the central region of Vietnam, and LAB diversity was assessed by quantitative real-time polymerase chain reaction (PCR) and qualitative denaturing gradient gel electrophoresis (DGGE). Differences among retailers and households were also examined.

## 2. Materials and Methods

### 2.1. Samples of fermented foods

Twenty-five samples with five products each of *dua gia* (bean sprouts), *dua cai* (cabbage), *mang chua* (bamboo shoot), *nem chua* (uncooked pork), and *tre* (cooked pork) were purchased from 12 shops at six local markets in Hue.

### 2.2. Chemical analyses

Dry matter content was determined by drying the materials in an oven at 60 °C for 48 h. The lactic acid, short chain fatty acid, and alcohol contents were determined from water extracts using an ion-exclusion polymeric high-performance liquid chromatography method with refractive index detection [5]. A portion of the water extracts was passed through a 0.20 µm filter, and 10 µL of the filtrate was injected into an ICSep COREGEL-87H column (Tokyo Chemical Industry Co., Ltd., Tokyo, Japan) containing a cation exchange polymer in the hydrogen ion form. The mobile phase was 0.004 mol/L sulphuric acid, and the flow rate was 0.6 ml/min at 60 °C.

### 2.3. DGGE

Samples were added to a 10× volume of sterilized phosphate buffered saline (pH 7.4). Extraction involved shaking the samples vigorously for 10 min and obtaining microbial pellets by centrifugation at 8000× *g* for 15 min. Bacterial DNA was purified using a commercial kit (Blood and Tissue Kit; Qiagen, Germantown, MD, USA), according to the manufacturer's instructions. PCR was employed to amplify a variable (V3) region of the bacterial 16S rRNA gene using primers and PCR protocols as previously described [6].

The PCR products were separated according to their sequences using a DCode Universal Mutation Detection System (Bio-Rad Ltd., Tokyo, Japan). The samples were applied directly to 100 g/L (w/v) polyacrylamide gels that had a denaturing gradient ranging from 25% to 50%; these gradients had been prepared using 7 mol/L urea and 400 mL/L formamide respectively, as 100% denaturants. Electrophoresis was performed at a constant voltage of 150 V for 12 h at 60 °C.

After electrophoresis, the gels were stained with SYBR Green (Cambrex Bio Science Inc., Rockland, ME, USA) and photographed under UV illumination. Select bands were excised from DGGE gels, and extracted DNA was amplified by PCR, followed by cloning into the pTAC-1 vector. The resulting plasmids were transformed into *Escherichia coli* strain DH5α competent cells (DynaExpress TA cloning kit; BioDynamics Laboratory Inc., Tokyo, Japan). The DNA base sequences were analysed using an ABI PRISM® 3130 sequencer (Applied Biosystems Inc., Foster City, CA, USA) and Basic Local Alignment Search Tool (BLAST) searches were performed against the GenBank database. The rRNA gene sequences determined in this study have been deposited in the DNA Data Bank of Japan (Accession Numbers LC205657–LC205687).

### 2.4. Real-time PCR assessment of bacterial populations

Amplification and detection of bacterial DNA were performed with KAPA SYBR® FAST qPCR Master Mix (Kapa Biosystems, USA) and a Mini Opticon real time PCR system (Bio-Rad Laboratories Inc., Tokyo, Japan). Primers targeting the 16S rRNA genes of the total bacterial population [7] and those targeting *Lactobacillus* spp. [8] were employed. Plasmid DNA, prepared with 16S rRNA genes of *L. brevis* (JCM 1059T), was used as a standard for qPCR analyses of both total bacteria and *Lactobacillus* spp. A three-step protocol was performed with an initial denaturation at 95 °C for 5 min followed by 35 cycles of denaturation at 95 °C for 15 s, annealing at 60 °C (total bacteria) or 58 °C (*Lactobacillus* spp.) for 20 s, and extension at 72 °C for 30 s. After the final qPCR cycle, melting curve analysis of the qPCR products was performed by heating from 60 °C to 95 °C, and the fluorescence signal was measured every 0.3 °C in the last 10 s to verify qPCR products.

### 2.5. Statistical analyses

Quantitative data were subjected to a one-way analysis of variance and a Tukey's multiple range test was used to examine the differences between food items (JMP ver. 11; SAS Institute, Tokyo, Japan). Qualitative DGGE band profiles were converted to a list of binary numbers using an image analysis system (Image J; NIH, Bethesda, MD, USA). A total of 39 bands were used in principal coordinate (PCoA) analysis on the Bray-Curtis similarities matrix using Primer version 7 with Permanova+ add-on software (Primer-E, Plymouth Marine Laboratory, Plymouth, UK).

## 3. Results

All samples were evaluated as being acceptable based on acidity and fermentation product composition (Table 1). Dry matter contents of vegetable-based fermented products were approximately 7 g/kg and those of meat-based products were 34–50 g/kg. *Dua gia* (bean sprouts), *dua cai* (cabbage), and *nem chua* (uncooked pork) had more lactic acid than acetic acid, whereas *mang chua* (bamboo shoot) and *tre* (cooked pork) showed the opposite trend. Ethanol was detected in one sample of *dua gia* (bean sprouts) at 3.9 g/kg but was not detected in all other samples. *Mang chua* had the lowest pH value among the five food items.

Total bacterial populations ranged from 1.9 × 10^9^ to 9.1 × 10^9^ copies/g and *Lactobacillus* spp. populations ranged from 1.5 × 10^9^ to 7.6 × 10^9^ copies/g. *Tre* (cooked pork) had higher LAB numbers than *dua cai* (cabbage) and *mang chua* (bamboo shoot). The proportion of *Lactobacillus* spp. to total bacteria was the lowest (77%) for *dua cai* (cabbage) and the highest (94%) for *nem chua* (uncooked pork).

**Figure 1.**
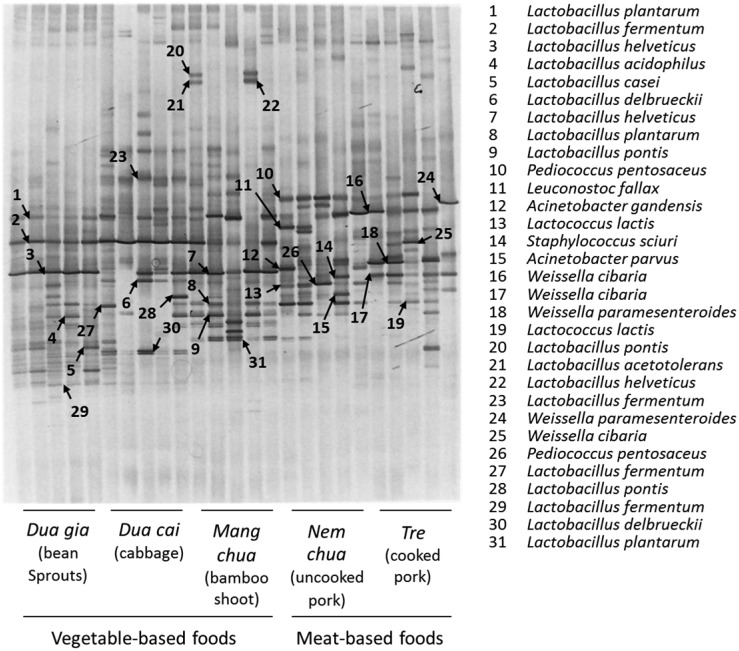
Denaturing gradient gel electrophoresis analysis of the microbiota of vegetable-based and meat-based fermented foods produced in the central region of Vietnam.

DGGE analysis demonstrated distinctive diversity of microbiota within and between vegetable-based and meat-based fermented foods. In vegetable-based products, bands corresponding to *L. plantarum* (band 1), *L. fermentum* (band 2), and *Lactobacillus helveticus* (band 3) were common to all three food items. However, despite the fact that all samples of *dua gia* (bean sprouts) resulted in clear bands representing *L. plantarum*, *L. fermentum*, and *L. helveticus*, several samples of *dua cai* (cabbage) and *mang chua* (bamboo shoots) did not have one or two of the three LAB species. Bands for *Lactobacillus delbrueckii* (bands 6 and 30) were clearly observed for *dua cai* (cabbage), and a band for *Lactobacillus pontis* (bands 9 and 20) was distinctive for *mang chua* (bamboo shoots). *Lactobacillus acidophilus* (band 4), *Lactobacillus casei* (band 5), and *Lactobacillus acetotolerans* (band 21) were occasionally detected in vegetable-based fermented foods.

**Figure 2.**
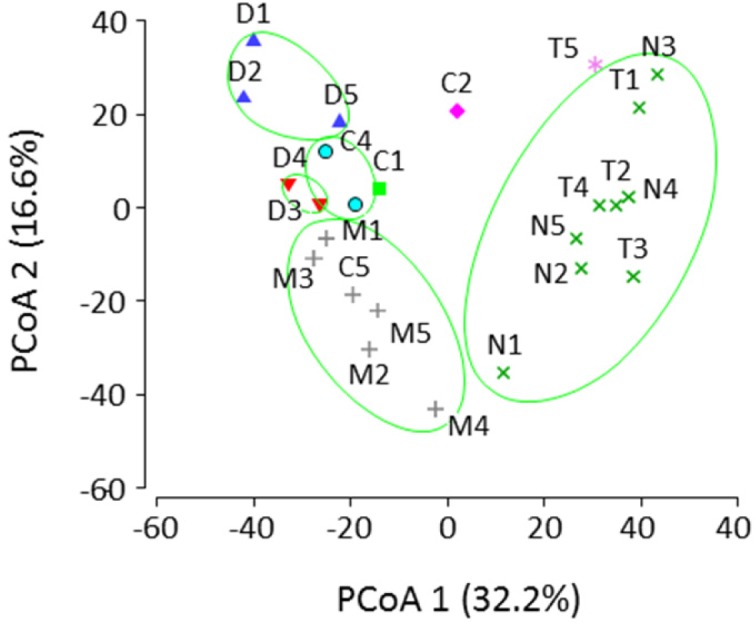
Principal coordinate analyses (PCoA) examining the differences in microbiota between vegetable-based and meat-based fermented foods produced in the central region of Vietnam. *Dua gia* (bean sprout), *cai chua* (cabbage), *mang chua* (bamboo shoots), *nem chua* (uncooked pork), and *tre* (cooked pork) are abbreviated as D, C, M, N, and T, respectively. The values in parentheses are the percentages of variation accounted for by the PCoA axes.

Band patterns from *nem chua* (uncooked pork) and *tre* (cooked pork) were largely different from those from *dua gia* (bean sprouts), *dua cai* (cabbage), and *mang chua* (bamboo shoots). Common bands for two meat-based products were *P. pentosaceus* (band 10), *Weissella cibaria* (band 16), and *Lactococcus lactis* (bands 13 and 19). *P. pentosaceus* was more often identified in *nem chua* (uncooked pork) and *W. cibaria* was more typically found in *tre* than in its meat-based counterpart. Other LAB species occasionally detected in *nem chua* (uncooked pork) and *tre* (cooked pork) were *Leuconostoc fallax* (band 11) and *Weissella paramesenteroides* (bands 18 and 24). In addition, several non-LAB species, namely *Staphylococcus sciuri* (band 14), *Acinetobacter gandensis* (band 12), and *Acinetobacter parvus* (band 15), were detected exclusively in *nem chua* (uncooked pork).

PCoA indicated that, although meat-based products were grouped together (except for one *tre* sample), vegetable-based products were classified into four small groups and additional separate clusters. Samples of *mang chua* (bamboo shoots) formed an independent group, whereas those of *dua cai* (cabbage) were seen as scattered plots. In addition to a clear difference between vegetable-based and meat-based products, significant microbiota diversity between households was shown for *dua cai* (cabbage).

**Table 1.** Acidity, concentrations of dry matter, lactic acid, and acetic acid, and real time PCR assessment of total bacterial number, *Lactobacillus* spp. number, and their relative proportion in vegetable-based and meat-based fermented foods produced in the central region of Vietnam.

	*Dua gia* (bean sprouts)	*Cai chua* (cabbage)	*Mang chua* (bamboo shoot)	*Nem chua* (pork)	*Tre* (cooked pork)
pH	4.33 ± 0.10^bc^	4.45 ± 0.16^b^	4.10 ± 0.06^c^	4.91 ± 0.13^a^	5.09 ± 0.33^a^
Dry matter (g/kg)	7.58 ± 0.37^c^	7.32 ± 1.26^c^	7.26 ± 1.96^c^	35.4 ± 1.26^b^	48.0 ± 6.73^a^
Lactic acid (g/kg)	4.20 ± 1.18^bc^	3.02 ± 1.13^c^	3.92 ± 1.00^bc^	13.1 ± 2.84^a^	8.37 ± 4.11^b^
Acetic acid (g/kg)	0.68 ± 0.40^c^	2.22 ± 0.77^bc^	4.46 ± 2.51^abc^	5.86 ± 3.22^ab^	9.25 ± 3.85^a^
Total bacteria (log copies/g)	9.67 ± 0.18^ab^	9.31 ± 0.17^b^	9.28 ± 0.36^b^	9.61 ± 0.17^ab^	9.96 ± 0.15^a^
*Lactobacillus* spp. (log copies/g)	9.63 ± 0.25^ab^	9.19 ± 0.19^b^	9.19 ± 0.43^b^	9.58 ± 0.22^ab^	9.88 ± 0.19^a^

Data are means ± standard deviations for five samples of each fermented food. Values in the same row with different superscript letters are significantly different (*p* < 0.05).

## 4. Discussion

Although selected starters were not used in any fermented foods, LAB were predominant, and populations were identified levels at 10^9^ copies/g in both vegetable-based and meat-based products. Similar levels of LAB (10^8−9^ cfu/g) were observed for Vietnamese fermented foods, regardless of the region, using MRS plates [3],[4]. Concentrations of dry matter and organic acids in *nem chua* (uncooked pork) were also similar to those reported by Yoshimura et al. [9]. However, the pH values of *nem chua* (uncooked pork) in this study were relatively high compared to those of other reports, wherein the pH value was found to decrease to as low as 3.8–4.1 after three to four days of fermentation [9],[10]. The pH and organic acid content between *nem chua* (uncooked pork) and *tre* (cooked pork) were similar, indicating that the use of cooked pork did not greatly influence the fermentation process.

Tran et al. demonstrated that, although *L. plantarum* was predominant in *nem chua* (uncooked pork) produced in the north and south of Vietnam, *P. pentosaceus* was mainly isolated in *nem chua* (uncooked pork) from the central region [4]. Our study using products from the central region of Vietnam also indicated that *P. pentosaceus* was frequently found in *nem chua* (uncooked pork).

*L. lactis* was steadily detected in *nem chua* (uncooked pork) and *tre* (cooked pork) in this study. Although Nguyen et al. found *L. lactis* in *nem chua* (uncooked pork), the proportion was as small as 1/119 isolates [11]. Because MRS medium enriches for *Lactobacilli* growth, *Lactococcus* spp. could be underrepresented using plate-culture compared to the use of culture-independent analysis. Indeed, Nguyen et al. detected *L. lactis* in *nem chua* (uncooked pork) by DGGE despite the fact that no *L. lactis* was identified in MRS isolates [3].

According to Nguyen et al., who examined 21 samples and 881 isolates by MALDI-TOF-MS and *pheS* gene sequence analysis [11], LAB including *L. fermentum* (56%), *L. pentosus* (24%), and *L. plantarum* (17%) were prevalent in vegetable-based products. Although *L. fermentum* and *L. plantarum* were also found, no *L. pentosus* was observed in this study, probably because *L. plantarum*, *L. paraplantarum*, and *L. pentosus* are difficult to discriminate by 16S rRNA gene analysis [12]. Meanwhile, our finding that *L. helveticus* was frequently identified in vegetable-based products was an interesting addition to previous knowledge. Likewise, *L. acetotolerans* and *L. pontis* have never been detected in Vietnamese fermented foods. *L. acetotolerans* and *L. pontis* are regarded as inhabitants of fermented foods with high acetic acid content, such as sourdough [13]. Because *mang chua* (bamboo shoot) had the highest acetic acid content among vegetable-based products, its detection in two samples was not unexpected.

Although clear differences were seen in microbiota between vegetable-based and meat-based products, it cannot simply be ascribed to the difference of the ingredients; microbiota involved in fermentation might have been affected by differences in dry matter concentrations. Although Tran et al. and Nguyen et al. found that *L. plantarum* was a predominant LAB species in *nem chua* (uncooked pork) [4],[11], no *L. plantarum* was identified in this product. Likewise, although Nguyen et al. isolated higher numbers of *L. farciminis* compared to that of *L. plantarum* from *nem chua* (uncooked pork) [11], *L. farciminis* was not detected in any samples in this study. Meanwhile, the finding of Nguyen et al. that *L. fermentum*, a prevalent LAB in vegetable-based products [11], was not identified in *nem chua* was in agreement with our results.

Our results that *W. cibaria* and *W. paramesenteroides* were often found in *tre* (cooked pork) were also of interest. Although PCoA indicated a similar group for *nem chua* (uncooked pork) and *tre* (cooked pork), bands for *W. cibaria* and *W. paramesenteroides* were more distinctive in *tre* (cooked pork). Nguyen et al. also detected *W. cibaria* and *W. paramesenteroides* in *nem chua* (uncooked pork), but the proportions were 2/119 and 1/119, respectively [11]. Because *Weissella* spp. are widely observed in fermented plant and animal foods [14], the distinct detection of *Weissella* spp. in *tre* (cooked pork) is difficult to explain. However, individuals in the central regions might have the opportunity to take in *Weissella* spp. through *tre* (cooked pork) consumption. The primer set for *Lactobacillus* spp. quantification by real-time PCR is able to detect *Weissella* spp., *Leuconostoc* spp., and *Pediococcus* spp. in addition to *Lactobacillus* spp.; hence, most LAB species detected by DGGE should have been appropriately quantified.

The presence of *Acinetobacter* sp. and *Staphylococcus* sp. in *nem chua* (uncooked pork) was also an interesting addition to current knowledge. Because the majority of previous studies have focused on the potential benefits of LAB, information on microbiota based on food hygiene is lacking. In addition to the screening and understanding of beneficial LAB species, research with the goal of promoting the quality of naturally fermented foods is required.

## 5. Conclusion

Microbiota analysis combining DGGE and real-time PCR was performed on vegetable-based and meat-based fermented foods produced in Hue, a city in the central part of Vietnam. Major LAB species found in *dua gia* (bean sprouts), *dua cai* (cabbage), and *mang chua* (bamboo shoots) were *L. plantarum, L. fermentum*, and *L. helveticus*, whereas those in *nem chua* (uncooked pork) and *tre* (cooked pork) were *P. pentosaceus*, *W. cibaria*, and *L. lactis*. Because the association between *L. helveticus*, *W. cibaria*, and *L. lactis* and these food products was newly depicted in this study, our results are a good addition to the current knowledge of fermented food products. Several indigenous LAB species could be used as starters to assure the hygienic quality of *nem chua* (uncooked pork) fermentation, which is regarded as unstable and occasionally disrupted by the development of undesirable microorganisms.
